# Supplementary material for: The genome sequence of the Cramp-ball Fungus Weevil, Platyrhinus resinosus (Scopoli, 1763) (Coleoptera: Anthribidae)
Source: Wellcome Open Res. 2026 Jun 2;11:327. [Version 1] doi: 10.12688/wellcomeopenres.26677.1 (PMC13309772; doi:10.12688/wellcomeopenres.26677.1)
Supplement: Peer review history

# Reviewer report: Reviewer response for version 1

**DOI:** 10.21956/wellcomeopenres.29378.r157522

**Published:** 2026-06-26

**Authors:** Rebecca Funari

**Affiliations:** 1 University of Siena, Siena, Italy

The manuscript by Crowley and Roman Maldonado, entitled The genome sequence of the Cramp-ball Fungus Weevil, *Platyrhinus resinosus* (Scopoli, 1763) (Coleoptera: Anthribidae), presents a high-quality chromosome-level genome assembly for *P. resinosus*. The manuscript is well written and well organized, providing an informative overview of the species together with a detailed and reproducible description of the sequencing, assembly, and quality assessment workflow. The assembly appears to be of high quality, with haplotype 1 scaffolded into 11 pseudomolecules and supported by the reported completeness and quality metrics. 

 I only have a few minor comments that, if addressed, would further improve the clarity and consistency of the manuscript:

 1) I suggest repeating the full scientific name *Platyrhinus resinosus* (Scopoli, 1763) at its first mention in the Background section.

 2) Please standardize the reporting of kit and instrument details throughout the manuscript. Manufacturer location (e.g., company, state/city, country) should be reported consistently, as country information is currently missing for several kits and instruments.

 3) Please define the abbreviation QV at its first mention in the text.

Are sufficient details of methods and materials provided to allow replication by others?

Yes

Is the rationale for creating the dataset(s) clearly described?

Yes

Are the datasets clearly presented in a useable and accessible format?

Yes

Are the protocols appropriate and is the work technically sound?

Yes

Reviewer Expertise:

Population genomics, Genomics, Mitogenomics, Entomology

I confirm that I have read this submission and believe that I have an appropriate level of expertise to confirm that it is of an acceptable scientific standard.

# Reviewer report: Reviewer response for version 1

**DOI:** 10.21956/wellcomeopenres.29378.r157519

**Published:** 2026-06-11

**Authors:** Rajib Deb

**Affiliations:** 1 ICAR-National Research Centre, Guwahat, Assam, India

The manuscript presents a high-quality chromosome-level genome assembly of *Platyrhinus resinosus* generated as part of the Darwin Tree of Life project . The sequencing and assembly protocols are appropriate and technically sound, and sufficient methodological details are provided to ensure reproducibility. The datasets are clearly presented and publicly accessible.

 The rationale for generating the dataset is only partly developed. Although the biological background is described, the significance of sequencing this species could be explained more clearly. In particular, the statement that the genome will provide insights into "venom composition" appears unsupported and should be revised or justified.

 Overall, this is a valuable genomic resource and I recommend indexing after minor revision. The authors should strengthen the biological rationale and clarify unsupported claims to improve the scientific context of the study.

Are sufficient details of methods and materials provided to allow replication by others?

Yes

Is the rationale for creating the dataset(s) clearly described?

Partly

Are the datasets clearly presented in a useable and accessible format?

Yes

Are the protocols appropriate and is the work technically sound?

Yes

Reviewer Expertise:

Genomics

I confirm that I have read this submission and believe that I have an appropriate level of expertise to confirm that it is of an acceptable scientific standard.
